# Accurate Prediction of Peptide Binding Sites on Protein Surfaces

**DOI:** 10.1371/journal.pcbi.1000335

**Published:** 2009-03-27

**Authors:** Evangelia Petsalaki, Alexander Stark, Eduardo García-Urdiales, Robert B. Russell

**Affiliations:** European Molecular Biology Laboratory, Heidelberg, Germany; Columbia University, United States of America

## Abstract

Many important protein–protein interactions are mediated by the binding of a short peptide stretch in one protein to a large globular segment in another. Recent efforts have provided hundreds of examples of new peptides binding to proteins for which a three-dimensional structure is available (either known experimentally or readily modeled) but where no structure of the protein–peptide complex is known. To address this gap, we present an approach that can accurately predict peptide binding sites on protein surfaces. For peptides known to bind a particular protein, the method predicts binding sites with great accuracy, and the specificity of the approach means that it can also be used to predict whether or not a putative or predicted peptide partner will bind. We used known protein–peptide complexes to derive preferences, in the form of spatial position specific scoring matrices, which describe the binding-site environment in globular proteins for each type of amino acid in bound peptides. We then scan the surface of a putative binding protein for sites for each of the amino acids present in a peptide partner and search for combinations of high-scoring amino acid sites that satisfy constraints deduced from the peptide sequence. The method performed well in a benchmark and largely agreed with experimental data mapping binding sites for several recently discovered interactions mediated by peptides, including RG-rich proteins with SMN domains, Epstein-Barr virus LMP1 with TRADD domains, DBC1 with Sir2, and the Ago hook with Argonaute PIWI domain. The method, and associated statistics, is an excellent tool for predicting and studying binding sites for newly discovered peptides mediating critical events in biology.

## Author Summary

An important class of protein interactions in critical cellular processes, such as signaling pathways, involves a domain from one protein binding to a linear peptide stretch of another. Many methods identify peptides mediating such interactions but without details of how the interactions occur, even when excellent structural information is available for the unbound protein. Experimental studies are currently time consuming, while existing computational methods to predict protein–peptide structures mostly focus on interactions involving specific protein families. Here, we present a general approach for predicting protein–peptide interaction sites. We show that spatial atomic position specific scoring matrices of binding sites for each peptide residue can capture the properties important for binding and when used to scan the surface of target proteins can accurately identify candidate binding sites for interacting peptides. The resulting predictions are highly illuminating for several recently described protein–peptide complexes, including RG-rich peptides with SMN domains, the Epstein-Barr virus LMP1 with TRADD domains, DBC1 with Sir2, and the Ago hook with the Argonaute PIWI domain. The accurate prediction of protein–peptide binding without prior structural knowledge will ultimately enable better functional characterization of many protein interactions involved in vital biological processes and provide a better picture of cellular mechanisms.

## Introduction

Protein–protein interactions are vital for all cellular processes, including signaling, DNA repair, trafficking, replication, gene-expression and metabolism. These interactions can vary substantially in how they are mediated. What perhaps most often comes to mind are interactions involving large interfaces, such as those inside the hemoglobin tetramer, however, many important protein interactions, particularly those that are transient, low-affinity or related to post-translational modification events like phosphorylation, are mediated by the binding of a globular domain in one protein to a short (e.g., 3–10 amino acid) peptide stretch in another [1]. These stretches often reside in the non-globular and/or disordered parts of the proteome, including many of the disordered interaction hubs [2],[3], thus helping to explain many of the emerging functional roles for such regions. Peptide regions binding to a common protein, or domain, often conform to a sequence pattern, or *linear motif* that captures the key features of binding [4]. For instance, SH3 domains bind PxxP motifs, WW domains bind PPxY or PPLP motifs, and SH2, 14-3-3 and PTB domains bind phosphorylated peptides [1]. Since they are generally held to be more chemically tractable than interactions involving larger interfaces, protein–peptide interactions also represent an important new class of drug targets, and there are a growing number of small molecules that are designed to target them [5].

The discovery of new peptides and motifs mediating interactions has been of intense interest in recent years (e.g., [6]–[8]). Several techniques have been developed to uncover new variants of peptides that bind to known partners. For instance, phage display and peptide array technologies have been applied to uncover new peptide partners for many proteins or domains, including SH3 [9], WW [10] and PDZ [11] domains. Several computational approaches have also been developed that use protein–peptide complexes of known 3D structure to find additional peptides that are likely to bind (e.g., [12]–[16]), and recently, probabilistic interaction networks have been used to predict peptide regions corresponding to kinase substrate [17]. The common thread to all of these approaches is that they rely on prior knowledge of the type of peptide binding to a domain and often require further knowledge of the peptide binding site on the globular protein. They are thus generally only effective for finding new variants of known peptides, and cannot directly uncover new protein–peptide interaction types. Protein–protein docking is currently the only widely used technique that can be applied to this problem generally, however this approach has limited application for peptides longer than 4 residues largely owing to the high degree of flexibility that one must consider when docking a typical peptide of 5–10 residues or the need for a known peptide conformation which is only rarely available [18]. Moreover, docking methods are very sensitive to conformational changes and require very high-resolution structures to perform well.

Determining new protein–peptide interaction types is problematic experimentally, mostly because it is difficult in advance to know the regions in larger proteins responsible for binding, necessitating painstaking experiments such as deletion mutagenesis coupled to binding assays (e.g., [6],[19]). To address this, several computational methods have been developed to discover new protein–peptide-motif pairs using the principle of sequence over-representation in proteins with a common interacting partner [6]–[8]. These methods, together with much conventional work focused on understanding interactions, have identified or predicted hundreds of new peptide-motifs mediating interactions with particular protein domain families. However, these discoveries rarely provide information about where the peptide binds the protein. Knowing these details can suggest further experiments and help ultimately to design chemical modulators of the interaction.

**Figure 1:**
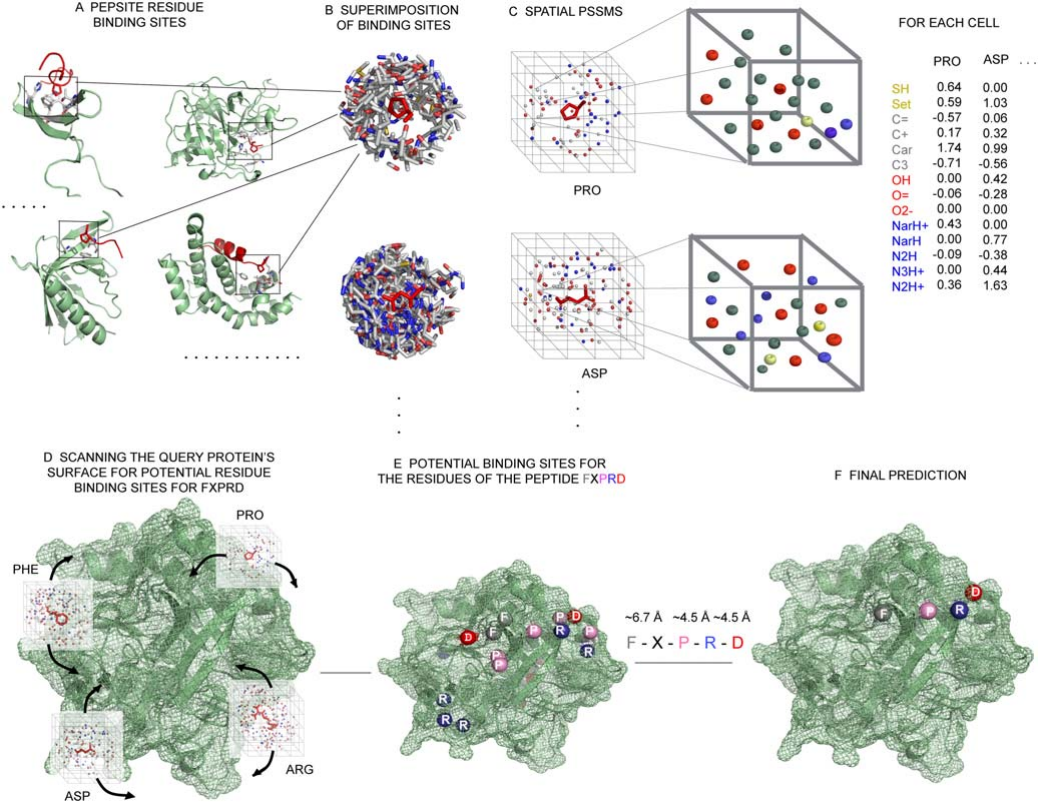
Overview of the method. (A) A training dataset of protein–peptide complexes is extracted from the Protein Data Bank [20]. (B) The peptide residues are superimposed along with their associated binding environments. (C) Spatial Position Specific Scoring Matrices (S-PSSMs) are created based on the spatial distribution of 14 defined atom types (Table S3) in the binding site of each residue. compared to background protein surfaces sites (D) S-PSSMs corresponding to residues in a query peptide (FxPRD) are then scanned over the surface of the protein. (E) Potential binding sites for each residue of the query peptide are identified, which are then combined using the distance constraints dictated by the peptide sequence. (F) The binding site for the complete peptide is predicted and scored.

Structures of protein–peptide complexes for all newly discovered interactions will require substantial time to become available, though the rapid increase in structural data for single proteins means that very often 3D structures are available (or readily modeled) for at least part of a protein in isolation. There is thus a widening gap between proteins of known structure that are known or predicted to bind to a particular peptide and available 3D complexes that would foster a deeper understanding of mechanism and afford the discovery of additional peptides. Here we present a method that attempts to bridge this gap by predicting the binding site for peptides on protein surfaces. We used a dataset of protein–peptide complexes of known 3D structure extracted from the Protein Data Bank (PDB) [20] to define spatial position specific scoring matrices (S-PSSMs) capturing preferences for how each amino acid binds to protein surfaces. Three dimensional position specific scoring matrices have been used in the past to predict protein folding [21], to assess the quality of structural models [22] or to predict the function of proteins based on the matches of these position specific scoring matrices to a new protein structure [23] and to identify protein surface similarities [24]. However, to the best of our knowledge, they have not been used to predict interactions in this way. For a new protein–peptide pair, we identify candidate peptide binding sites by linking predicted sites for each residue on the protein surface according to peptide-deduced distance constraints (Figure 1). We developed statistics to determine the confidence of a prediction to estimate whether or not a putative peptide binds. When applied to a benchmark in a cross-validated fashion, we obtained excellent sensitivity and specificity, which allowed us to apply the approach to several new interactions, such as the interaction of the viral oncoprotein latent membrane protein 1 (LMP1) with the tumor necrosis factor receptor 1-associated death domain protein (TRADD) [25] offering suggestions of binding sites for further investigation.

## Results

### Spatial Position Specific Scoring Matrices Capture Amino Acid Binding Site Preferences

We created spatial position specific scoring matrices (S-PSSMs) for each of the 20 standard amino acids and three phosphorylated variants to capture their preferred binding environment. We superimposed the binding sites for each type of amino acid and quantified the protein atom preferences in a 3D grid (see Materials and Methods). Comparing S-PSSMs between amino acids shows that those with similar properties are often bound to similar binding sites, as might be expected (Figure S1) with certain exceptions (e.g., Trp/Gly). For example, S-PSSMs for phosphorylated amino acids are similar to glutamate or asparate, but differ from that for positively charged arginine (Figure S1). We then used the S-PSSMs to scan protein surfaces to predict binding sites for amino acids and, based on distance constraints between them, binding sites for peptides (see Materials and Methods). Figure 1 shows an overview of how the S-PSSMs are generated and how searches for binding sites are performed.

### Performance on Benchmark Datasets

To assess the performance of the method in its ability to identify the correct binding sites for peptides, we constructed a large benchmark of 405 known protein–peptide complexes (Dataset S1), from our training set, where at least one structure of the protein not bound to the peptide was available (see Materials and Methods). We then predicted binding sites for all peptides in the set to all corresponding un-bound protein structures using leave-one-out cross validation to ensure that no information derived from identical or homologous proteins was used to compute the parameters. Additionally we predicted the binding sites of random peptides of variable length to random chains from the structure database assuming this to be our negative dataset. For an additional benchmark, we extracted a smaller dataset of 18 protein–peptide complexes that were deposited in the PDB after we had constructed our training dataset (i.e., after 1st March 2007), and for which we could find the corresponding un-bound protein, and where these did not have a sequence similar to any protein used in the larger benchmark. The rationale was that this would provide a true test of the approach, since none of the development of the method could be biased in any way by exposure to these new complexes.

**Figure 2:**
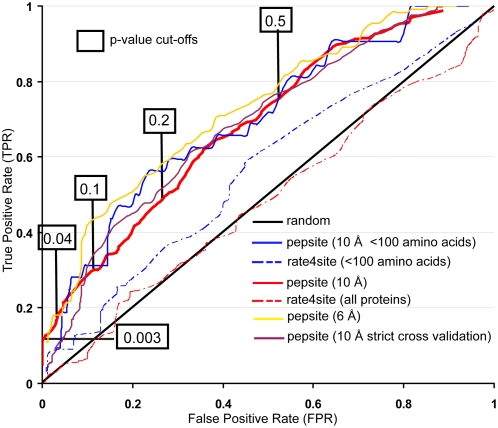
ROC curve showing performance in the large benchmark. False positive rate (X axis) plotted against true positive rate (Y) for different p-value cut-offs. False positive predictions are defined as those that either have predicted the wrong binding site or have predicted a binding site for a peptide that is not known to bind. The figure shows the result for our approach (pepsite) at two distance thresholds defining accuracy (6 Å & 10 Å), and for 10 Å with a subset of proteins smaller than 100 amino acids. Equivalent values for rate4site on the same datasets are also shown as well as the ROC curve for pepsite using a stricter cross-validation (i.e., excluding similarities/homologies between proteins as given in the SCOP database).

The ROC curve (Figure 2) shows the false positive rate (x-axis) versus the true positive rate (y-axis) when varying the p-value cutoff and testing whether a peptide, predicted to bind to a site was correct (i.e., a true positive) or incorrect (a false positive). The ratio of the true positive predictions to the total number of predictions made, represents the prediction accuracy of the method at different p-value cutoffs, i.e., it shows what fraction of the predictions made are actually correct and this corresponds to the statistical measure of positive predictive value (PPV). We used the top 5 scoring predictions for both the positive (1109 scores – we used only correct predictions) and negative dataset (2455 scores). The ROC curve, for the cross-validation tests on the large benchmark, also shows that the method performs well. For instance, predictions with p-values below 0.1 give a false positive rate (fraction of non-binding events wrongly predicted) of 0.1 and a true positive rate (fraction of known binding sites predicted correctly) of approximately 0.3 (295/1109), i.e., a PPV of 75%, while even a very low false positive rate of 0.01 (p-values below 0.003) still has a true positive rate of approximately 0.1 (94/1109), which represents a PPV of 89.9%. The Matthews correlation coefficient suggests the optimal p-value cut-off to be 0.04, which gives a false positive rate of 0.03, a true positive rate of 0.17 (186/1109), and a PPV of 85%. We obtain a similar result for the smaller benchmark, with statistically significant predictions (p<0.04) of the correct binding site for 2/18 (true positive rate of 0.11) complexes.

Overall, the ROC analysis suggests that the approach will correctly identify whether a peptide binds and where it binds for a reasonable number of peptide binding sites with significance. The curve resembles those for remote homology detection by techniques like PSI-blast [26] or threading when tested on difficult benchmarks consisting of structurally similar but sequence dissimilar proteins (e.g., [27]). This suggests that the problem of identifying binding sites in this way is a difficult one, but that the method can often nevertheless make useful predictions.

Although the tests above show a coverage of only about 11% for the optimal p-value, it is very important to emphasize that the ROC analysis tests the most difficult scenario, whereby one knows neither if a peptide binds nor where it binds to the protein surface. This neglects the common situation where one has identified a protein–peptide binding event, and does not know where on the surface it binds. To test this situation one must simply ask how often the correct binding site is found with any p-value. For the large benchmark this greatly increases the coverage: the correct site was found for 60% (241/405) of the complexes, which corresponds to the accuracy of the binding site prediction, with a similar fraction for the small benchmark (11/18).

Our leave-one-out cross-validation, which removed any detectable (BLAST [28] E-value<0.1) sequence homologues before evaluation, still leaves a possibility that remote homologues could in some way lead to S-PSSMs over-learning peptide binding sites. To remove this possibility, we repeated the benchmark process using stricter definitions of homology by taking single representatives from groups as defined in the Structural Classification Of Proteins (SCOP [29]) database (family, superfamily and fold). This gave results similar to those seen in the original benchmark: all redundancy reductions (SCOP family, superfamily and fold gave similar datasets) led to 56% (192/342) correct peptide binding site predictions and a ROC performance similar to that for the original dataset, which suggests that there was no real bias in the creation of the S-PSSMs even with the sequence only reduction. The similarity between family, superfamily and fold reduced sets is due to the fact that the vast majority of the remotely homologous relationships involving similar peptide binding sites are removed at the family level (e.g., all SH3 or WW domains are in the same SCOP family).

For very low p-values the method does not perform as well; this is because there are very few high scoring predictions left after removing proteins lacking SCOP assignments (i.e., the newest structures), and is not statistically significant. For example for p-values<0.003 only 6 of the 23 complexes that scored high in the original dataset are left in the stricter dataset thus reducing the true positive rate while not changing the false positive rate. Remote homologues can play some role in defining binding sites for each other—for instance in creating the original cross-validated S-PSSMs for proline, three distantly related WW domains (i.e., PDB IDs 1i5h, 1djyI and 1f8a) were present—but this effect does not appear to bias the overall performance.

### Failures in the Benchmark

For most unsuccessful predictions within the benchmarks (i.e., where the binding site was not predicted even with poor p-values) there are explanations for failure. For 32 (∼20%) out of the 165 incorrect predictions the peptide was bound via augmentation of a beta-sheet [30], with a strong influence of backbone interactions that are not currently considered because they are not based on the specificity of particular residues for specific binding sites which is the assumption the method is based on. In principle, this binding mode could be accommodated by considering a backbone profile and stricter distance constraints to enforce this conformation. For 25/165 (∼15%), the peptides adopted a helical or circular structure, making distance constraints less effective, and for 20/165 (∼12%) the peptide contained heteroatoms or modified residues (e.g., biotinylated lysines, etc.) that the method had not been trained on. There are currently too few examples of known structure to derive effective S-PSSMs for rare modifications.

**Figure 3:**
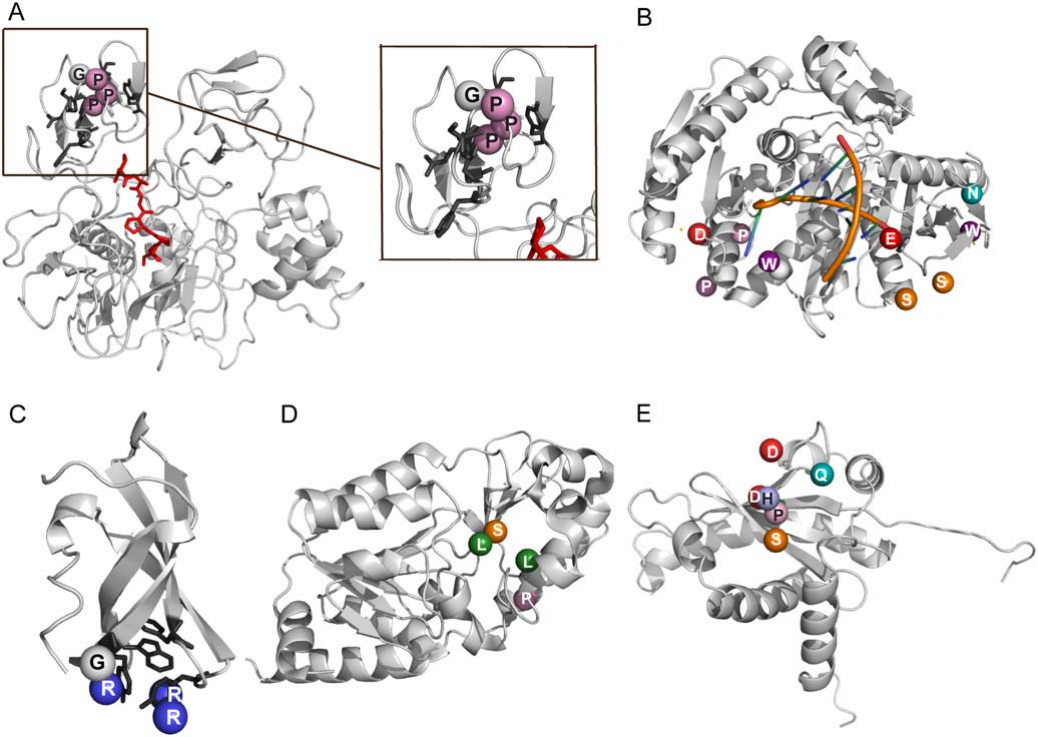
Examples of applying the method. Predicted peptides are depicted as spheres on the protein surface colored by amino acid type (prolines – pink, alanines and glycines - white, serines - orange, asparagines and glutamines - teal and aspartic/glutamic acid – red). (A) Binding of a collagen peptide (GPAGPPGA) on a human matrix metalloproteinase 2 (1eak). The peptide bound in the solved X-ray structure is colored in red. Note the predicted binding site differs however it is likely correct (see text). (B) Binding of the Ago hook peptide (PDNGTSAWGEPNESSPGWGEMD) on the PIWI domain of the Argonaute protein (PDB IDs: 1ytu [38]; 1w9h [39]): i) the best, though incorrect binding site; ii) the location of the other top scoring predictions (correct). (C) Prediction for the binding of an RGRGRGRG peptide to the human SMN tudor domain (PDB ID: 1mhn [40]), which agrees with NMR data. (D) Prediction of the leucine zipper (helical region 243–264) of the DBC1 sequence binding site on the catalytic domain of SIRT1 (PDB ID: 1m2g [42]) (E) Prediction for the binding of the LMP1 protein of the Epstein-Barr virus peptide DDPHGPVQLS on the TRADD protein (PDB ID: 1f2h [45]).

For 33/165 (∼20%) of the wrong predictions, we could see no obvious trends, but we noticed upon inspection that some were likely correct binding sites not seen in the complex structure. For example we predicted a different binding site for the peptide GPAGPPGA from that found in a complex with the human matrix metalloproteinase 2 (MMP2; PDB ID: 1eak). This unpublished structure appears to be a complex between MMP2 and a fragment of collagen/gelatin, the natural substrate (e.g., [31]). Our binding site does not agree with that in the complex, which resides in a central cavity of the protein, but is instead inside an exposed aromatic surface on a fibronectin domain (Figure 3A). This surface resembles that for many other proline-rich peptide binding proteins (e.g., SH3, WW, etc.). This was originally suggested to be the binding site for gelatin, based on an early single domain structure [32] and alanine scanning mutagenesis in MMP9 [33] and subsequent studies in MMP2 itself [34], showed that residues equivalent to our prediction were important for gelatin binding.

### Comparison to Predictions Based on Surface Conservation

Though no current approaches focus specifically on the problem of generally predicting peptide binding sites on protein surfaces, it is possible to predict binding sites generally by looking for patches of conservation on protein surfaces, an approach that has been under much focus for the past ten years [35]. Though not directly comparable, we applied one readily available algorithm, rate4site [36] to the same dataset for comparison. When considering the best predictions, conservation alone identifies 51% of peptide binding sites compared to 60% for our approach. However, the ROC curves show that conservation alone performs poorly in terms of specificity, owing largely to the fact that the approach identifies additional binding sites that do not bind peptides (Figure 2). Inclusion of the conservation of the binding sites in our predictive method results in a slight reduction of the coverage of binding sites being predicted with an improvement of only 2% in the true positive rate. Additionally it is computationally costly and it is only applicable for proteins that have a sufficient number of homologous sequences.

### Application to Recent Discoveries of Protein–Peptide Binding

We sought recently published examples of protein complexes distinct from those used in the benchmark to test the approach in a more real-world situation. Several recent protein–peptide complexes lack a 3D structure, but the location of the binding site has been partially determined by other means. For instance, the conserved linear motif termed the “Ago hook”, which was determined to bind to the PIWI domain of the Argonaute protein at the site where the 5′ end of an siRNA normally binds [37]. The interaction is important for transcriptional gene silencing and miRNA-mediated translational silencing, as well as for the recruitment of Ago proteins to specific cellular locations such as P-bodies. There were no available structures of Eukaryotic PIWI domains, so we predicted binding of the peptide PDNGTSAWGEPNESSPGWGEMD to Archaeal structures, either in isolation or bound to RNA (PDB IDs: 1ytu [38]; 1w9h [39]). Most of the best predictions lie near to the site of RNA binding (Figure 3B). A similar example is found in the tudor domain of the protein SMN, which plays a role in assembly of the spliceosomal ribonucleoprotein complexes by interacting with RG rich C-terminal tails of Sm proteins. NMR titration showed that these repeats bind on the tudor domain in a particular region rich in aromatic residues [40]. Our prediction for the binding of an RGRGRGRG peptide to the human SMN tudor domain (PDB ID: 1mhn [40]) matches the NMR mapped binding site (Figure 3C).

A recent example of a known protein–protein interaction delineated to a region in one protein binding another, but lacking a 3D structure, is the binding of the leucine zipper domain from Deleted in Breast Cancer-1 (DBC1) to the catalytic domain of the mammalian protein deacetylase Sir2 [41]. The predicted binding site on Sir2 (PDB ID: 1m2g [42]) lies in the same region as a p53 peptide (PDB ID: 1ma3 [43]; Figure 3D), and is thus consistent with the finding that DBC1 blocks the ability of Sir2 to deacetylate p53 [41]. A similar picture emerges for the binding by Tumor necrosis factor-receptor-1-associated death domain protein (TRADD) to Latent membrane protein 1 (LMP1) of the Epstein-Barr virus [44]. The 16 C-terminal residues of the LMP1 (GDDDDPHGPVQLSYYD) bind to the TRADD protein and cause the blockage of the apoptotic pathway, and induce the NF-kappaB pathway by recruiting and activating I-kappaB kinase beta [25]. The predicted binding sites on the TRADD N-terminal domain (PDB ID: 1f2h [45]) for 3 overlapping 10 residue peptides from this segment (GDDDDPHGPV, DDPHGPVQLS, and HGPVQLSYYD) are roughly in the same site (Figure 3E) as the TRAF2 protein (PDB ID: 1f3v [46]), which suggests the virus might affect apoptosis and other processes by mimicking the TRADD/TRAF2 association and subsequent binding to the kinase [25].

**Figure 4:**
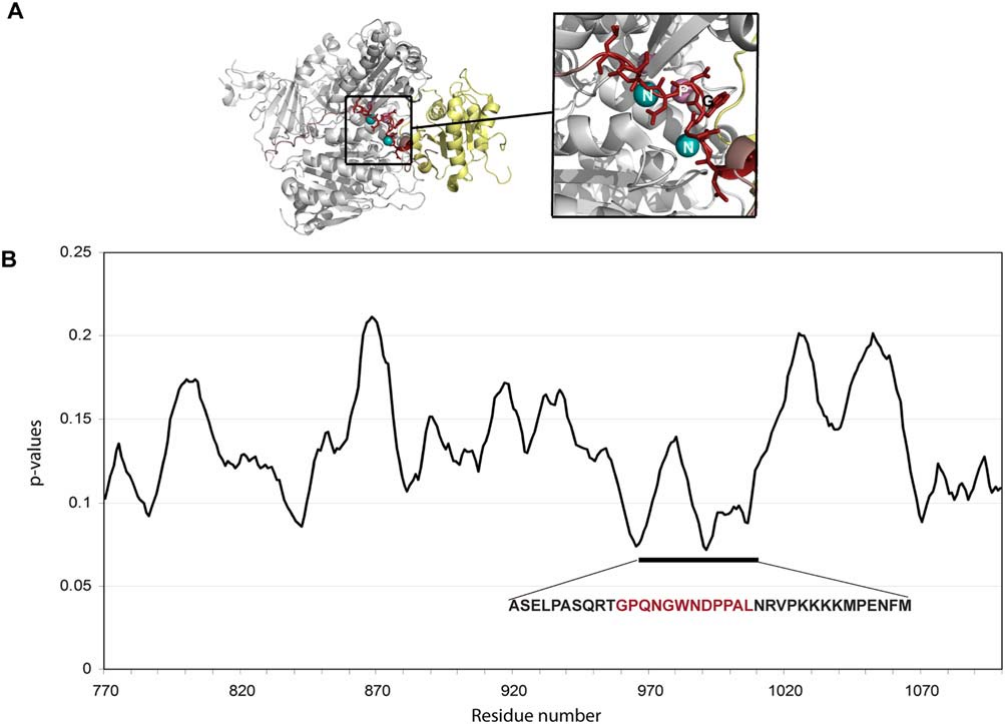
Using the method to scan for regions in Sec31 likely to bind Sec23. (A) Predictions for the most conserved region of the Sec31 disordered 40 residue peptide segment (GPQNGWNDPPAL) on the Sec23/Sar1 complex. In red is the region of the peptide from the solved structure (PDB IDs: 2qtv [19], 1m2o [48]). (B) P-values (Y-axis) for each 12 residue peptides from residues 770 to 1100 of the Sec31 protein (X-axis) to identify the binding region. The lowest p-values, in the region 965–1010, are very close to the known binding site (981–1021). The black line under the graph shows the actual binding 40 residue peptide and the region colored in red-brown corresponds to the peptide predicted to bind shown in (A) of this figure.

When a protein–protein interaction is known, but the regions involved are either not delineated, or are too long to be considered short peptides, our approach can be used to scan for putative binding peptides, by searching for significant scores among overlapping predictions within a region (or the entire protein). We demonstrate this for the interaction of Sec23/Sar1 with Sec31 which occurs as part of the COP II Coat Nucleation complex formation process [19]. A fragment of Sec31 (residues 850–1175) was initially identified to interact with full length Sec23 in a two-hybrid analysis [47]. This region largely overlaps with a proline-rich, disordered region that was subsequently revealed to contain a 40-residue segment responsible for the interaction, and confirmed by X-ray studies [19]. We scanned the region 770–1100 from human Sec31 (Uniprot O94979) using a 12 residue window for peptides that were predicted bind the Sec23/Sar1 complex (un-bound PDB ID: 1m2o [48]). The plot of averaged p-values (Figure 4B) shows the best peptides to be near to those known to bind Sec23/Sar1, and overlap with the most conserved region of the 40 residue region of Sec31 (Figure 4A).

## Discussion

This approach will be of benefit to researchers investigating the structural basis of protein–protein interactions. It can be applied to structures known to bind a peptide, and is likely to be informative about the site of interaction, and thus readily suggest further experiments to test the interaction. Although a lack of data currently prevents many modified residues from being studied we expect that the steady growth in structures will permit additional residues to be considered in the near future, and that new structures will continue to improve each residue profile and the approach. Additional data will ultimately permit more sensitive S-PSSMs, such as residue pairs, which we expect will greatly increase the performance. We are also currently developing modifications to account for the limitations mentioned above, such as the special case of peptides that bind via beta-sheet augmentation.

The method has advantages over many others that predict protein–peptide interactions. First, it does not require a known binding site, such as those approaches specifically tailored to predict SH3 or MHC binding peptides, and can thus be applied to any protein for which a structure is available and ideas about binding peptides or proteins. Second, it does not require that a substantial number of interactions be known for predictions to be made, but can in principle work on a single known or predicted peptide sequence. Most importantly, the method is accompanied by a statistic measure to estimate the reliability of predictions, which means it can be applied to many structures systematically to identify the strongest predictions, and to make predictions as to whether binding occurs at all.

Our approach partly systemizes what structural biologists often do when trying to guess a binding site from a protein surface (e.g., [49]) by trying to match properties of a binding peptide with complementary properties on the protein surface. However, it has the advantage that these inferences are coupled to rationally derived knowledge of how amino acids in peptides bind proteins, and a measure of the probability that such a predicted binding site might occur by chance. As such, it provides a more reliable starting point for site-directed mutagenesis, or other studies designed for finding true binding sites experimentally. It also provides an excellent starting point for protein docking approaches, which always fare better when applied to restricted binding regions instead of the entire protein surface. The fact that several sites are also found by a surface conservation method is perhaps not surprising, since proteins that bind peptides will undoubtedly often show conservation of the peptide binding site, as is generally true for all sites of molecular recognition. However, the improved performance over such approaches indicates that this method offers a more precise, and specific way to study peptide binding sites as distinct from general functional sites. Moreover, despite the fact that when attempting to directly combine the two approaches the improvement in accuracy is marginal and the cost in coverage is high, they can still be complementary: if a predicted binding site is also conserved this can provide additional evidence to increase confidence in a prediction.

As the number of known protein–protein interactions grows, so do the number of instances for which a peptide stretch is discovered to mediate an interaction of importance. At the same time, the increased pace of structure determination of single proteins or domains, means that it is now rare to find globular domains lacking structural information. Taken together, this suggests that techniques like that described here will be of growing importance to those interested in understanding, targeting and modifying protein interaction networks involved in critical biological processes.

## Materials and Methods

### Dataset

To train and test our method we created a manually-curated, non-redundant set of protein–peptide complex 3D structures. We first extracted 5055 complexes from the Protein Data Bank [20], in which peptide stretches of 3–20 residues were in contact with globular domains. Inspection showed that many complexes were due to non-specific crystal contacts. We corrected for this by manually inspecting a smaller subset of 386 highly non-redundant complexes (permitting only one member of any family from the SCOP database [29]), and classified these as one of: (1) true protein–peptide complexes; (2) protein–protein interactions mediated by a peptide stretch in one partner; and (3) probable crystal contacts. Within the first two categories, 85% of complexes had more than 18 protein atoms within 6 Å of those in the peptide, compared to only 20% in the crystal contacts set (Figure S2). We then applied this cutoff to the larger dataset to leave 2970 complexes.

We grouped the remaining complexes into 23 overlapping sets according to the amino acids contained in the peptide. Each set contained all complexes of proteins with a peptide stretch containing at least one of each particular amino acid (including the 20 standard plus phosphorylated serine, threonine and tyrosine). To derive spatial position-specific scoring matrices (S-PSSMs) for each amino acid (see below) we required the set of complexes corresponding to each amino acid to be non-redundant in order to avoid any bias due to homology. In principle, this could have been done across the entire dataset, however this lead to too few data points for the rarer amino acids (e.g., Trp, Met, phosphorylated-Tyr), owing to single complexes containing an amino acid in a peptide being removed because of homology to other complexes lacking it.

To make each set non-redundant, we performed an all against all BLAST sequence comparison [28] within each set and kept one representative of group of homologues sharing pairwise E-values< = 0.1. We selected preferably recently determined, refined X-ray structures, with the best resolution. These were then manually inspected to remove complexes that were due to crystal packing effects not captured by the filter above or that had missing residues, or instances where a presumed peptide was actually part of the original chain. This left a total of 553 complexes belonging to 364 SCOP families, in 23 sets for each amino acid. The number of non-redundant complexes per amino acid set varied from 13 for phosphorylated threonine to 288 for leucine (Table S1). Given that the redundancy reduction was performed inside each of the residue sets the full set of 553 inevitably contained some redundancy. For leave-one-out cross-validation, we thus removed a particular complex and its homologous representatives (as defined above) from every set in which it was contained, meaning that no similar complex would be used to construct the S-PSSMs. We also repeated the procedure using three stricter levels for the definition of homologous representatives, i.e., we removed all members of the same SCOP family, superfamily and fold for the leave-one-out cross-validation. Note, however, that all three levels gave almost identical datasets, since all cases of proteins binding peptides were similar at the family level, even if homology was remote (thus explaining a single curve in Figure 2).

### Construction of Spatial Position Specific Scoring Matrices

We created S-PSSMs for each of the 23 residues, capturing their preferred binding environment when present in a peptide. We first computed the solvent accessible atoms for each protein, having first removed the complexed peptide, using NACCESS [50] with default parameters, and kept only atoms with accessibility scores above zero. Our reasoning was that peptide stretches bind mostly to the surface of the protein and thus the solvent accessible surface should be sufficient to create robust matrices.

We then superimposed each of the residues found in the peptides, along with their associated protein environments. The superimposition is made in such a way that the residue side-chains are oriented the same way (Figure 1B). This way we could observe and quantify preferences for parts of each residue to be near to particular protein atoms in three dimensions. We first defined active parts of each side-chain as those most commonly involved in side-chain functions (i.e., the active center of each residue side-chain). We then performed superimpositions of the active part of each side chain using the PINTS [51] & STAMP packages [52]. For simplicity we did not consider all atoms of each side chain for the superimposition, but instead defined a subset (Table S2) that was sufficient for the PINTS & STAMP packages to obtain reasonable superimpositions.

To quantify the protein atom preferences for the space around each peptide residue r we created a grid over each superimposed residue environment. We placed the center of mass of the active part of each residue in the centre of the grid, and divided the space +/− 6 Å around it using a cell spacing of 3 Å (Figure 1C). We then studied the types of atoms found in each grid point, and computed a score for the preference of each atom type (as we have defined them based on their properties in Table S3) as:
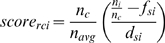
where *n_c_* is the number of atoms in cell c, *n_avg_* is the average number of atoms in a cell of this grid, *n_i_* is the number of atoms of type i in cell c, *f_si_* is the background frequency of atom type i on protein surfaces and *d_si_* is the standard deviation of the frequency of atom type i on protein surfaces. In theory these values can range from very negative, where the environment is very different to the one favored by the particular residue, to very positive, which represent a good match for the residue's binding site. For the best 10 sites on the protein surface that we define as hot spots (see below), the values are between −2 and 83 (Table S4).

### Prediction of Amino Acid Binding Hot Spots

To predict binding sites for a given peptide on a protein surface we first identify potential binding sites for each residue (hot-spots) by scanning and scoring the whole protein surface using the S-PSSMs (Figure 1D). To do this we place the corresponding S-PSSM at a specific distance from multiple planes defined on the protein surface, and oriented so that the active centre of the side chain faces the surface as if it were bound as a peptide residue on that protein site. This is accomplished by placing the centre of a grid on a vector perpendicular to a local plane centered at the surface atom and searching for the appropriate orientation of the grid. The distance is defined from our training dataset as the average of the minimum distances for each residue from the protein surface (Table S5). The planes are defined by two vectors starting at the atom for which we are calculating the score and ending at the previous (vector 1) or the next atom (vector 2) in the coordinate file. We assume that these atoms are close enough to the central atom to be able to define a valid local plane for the score calculation. In practice, this means that each amino acid is placed thousands of times on a structure in many different relative orientations (i.e., using each atom on the surface of the structure), and whilst it does not amount to a full 3D search, we found that it is more than adequate to sample the orientations actually found in known protein–peptide complexes. The procedure is roughly equivalent conceptually to rotating the protein with respect to the S-PSSM (Figure S3). In combination with the flexibility provided by the size of the S-PSSM cells (3 Å), this ensures that an effective sample of S-PSSM/protein orientations is considered when scanning the protein surface.

It is important to underscore that the method is not designed to detect precise atomic details of protein–peptide binding sites, but to offer approximate locations. This purpose is well served using this approximation, with the advantage that it saves on the computational time needed for an exhaustive 3D search of orientations of the peptide residue on every possible site of the protein.

The score for each orientation is calculated as:
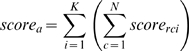
where K is the number of atom types that have been matched in the grid that was placed locally, N ( = 64) is the number of cells in the grid and *score_rci_* is the value from the S-PSSM of the particular residue r in cell c for atom type i. It is important to note that this procedure, i.e., orienting the S-PSSM appropriately and scoring the protein surface site, is performed for all atoms of the protein surface, thus ensuring a complete search of the space of possible surface/residue orientations.

### Prediction of Peptide Binding Sites

After scoring each site on the protein surface for each of the 23 amino acid S-PSSMs, we use the top ten scoring sites as potential binding sites for these residues (Figure 1E). It is possible to marginally improve the sensitivity of the approach by including more sites (i.e., down to a statistical significance threshold), but in practice this slowed the approach and hindered usage. We then search for combinations of amino acid hot-spots that are spaced such that they satisfy the constraints deduced from the peptide sequence. To derive the constraints we analyzed all peptides inside the training dataset to compute average distances between C-alpha atoms (D_Cal_) at particular sequence separations (i) and average distances from C-alpha atoms to the residue active centers (D_r_). Combinations of predicted residue sites are kept if all distances lie within D_Cal_±D_r_. In practice, these constraints are very flexible, with a slight preference for extended peptide conformations, since they grow as a function of sequence separation, and thus slightly disfavor helical or circular peptide conformations.

For each potential peptide match (Figure 1F) we calculated the overall score as:
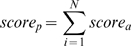
Where score_α_ is calculated using the formula above.

We then computed a statistical significance p-value for each score as 1−Φ(*x*) where Φ(*x*) is the cumulative distribution probability that represents the probability that a random variable V with that distribution is less than or equal to x. Therefore the p-value represents the opposite, i.e., the probability of the event that a random variable V with that distribution is more than or equal to x.

We calculated a background score distribution defining random scores as those for peptides selected randomly from our training dataset having fewer than 2 residues (in any position) in common with that seen to bind a particular protein. We selected 5 random peptides for each of the 405 un-bound proteins (see above). We cannot rule out that some of these random peptides will, in fact, bind to the proteins, but the statistics hold (and indeed will be conservative) even if a small fraction of random values correspond to positives.

We defined correct binding site predictions as those where the average distance between predicted and known amino acid locations was less than a threshold (between 6 and 10 Å). Visual inspection of several dozen predictions suggested 10 Å to be a reasonable upper limit, allowing for typical deviations in side-chain placements that occur after structural rearrangements upon binding, but not counting wildly different binding sites as correct.

### Comparison with Conserved Functional Site Predictions

We compared our method to the rate4site [36] program that predicts functional sites on proteins by finding clusters of conserved residues. To do this we ran PSI-Blast [26] using the sequence of the bound structure against the NCBI non-redundant databases. For those with at least 3 significant sequence matches, we created alignments of the best 50 sequences (which is the default for Consurf, the web version of rate4site) using ClustalW [53] and gave these (and the structure) as input to rate4site. We defined correct predictions in a lenient fashion as those where at least one of the top 5 conserved positions was within 10 Å of the bound peptide. For the ROC analysis we defined negatives as all other sites on the proteins. The results were very similar when using conservation scores calculated only for the solvent accessible residues and when using those for the full protein sequence. We therefore used the full protein sequence since this is the way the program is actually used.

### Availability

A server to run predictions using the PEPSITE approach is available at http://pepsite.embl.de.

## Supporting Information

Dataset S1 Benchmark dataset used for the evaluation of the method.
(0.01 MB TDS)

Figure S1 Comparison of S-PSSMs for the 20 standard amino-acid residues and phosphorylated tyrosine (PTR), threonine (TPO), and serine (SEP). Similarities increase from blue to red.
(3.25 MB TIF)

Figure S2 Graph to distinguish protein-peptide interactions from probable crystal packing effects. Distribution of the number of protein atoms within 6 Å of those in the peptide for the three categories: (1) protein-peptide complexes; (2) protein-protein interactions where the interaction consists largely of a peptide stretch from one binding to a globular segment in the other; and (3) probable crystal packing artifacts.
(0.75 MB TIF)

Figure S3 The algorithm for scanning and scoring protein surfaces using the S-PSSMs. (A) Example of real orientation for proline within a peptide relative to a phenylalanine residue on a protein surface. (B) Definition of orientation for the S-PSSM. For each atom of a protein surface a plane is defined using the atoms before and after it in the coordinate file. A vector of distance Dr is then defined to be perpendicular to this plane. (C) The S-PSSM is placed as defined by the previous vectors. (D) Examples of planes resulting to different orientations of the S-PSSM relative to the protein. (E) In practice and in combination with the flexibility provided by the grid cell size (3 Å) repeating this procedure for all protein surface atoms results in an effective rotation of the protein with respect to the S-PSSM in thousands of orientations.
(3.77 MB TIF)

Table S1 Number of complexes per amino acid dataset.
(0.03 MB DOC)

Table S2 Atoms from the active center of each peptide residue, used for the superimposition of the peptide residue binding sites. TYS was considered equivalent to PTR. Atoms marked with a * represent atoms are not part of the active site but used because the software requires minimum 3 atoms to perform the superimposition
(0.04 MB DOC)

Table S3 Atom types and their corresponding atoms in the coordinate files. The different atom types were generated based on their properties.
(0.03 MB DOC)

Table S4 Range of scores per residue hot-spots. The table shows minimum value, average value, standard deviation, and maximum value observed for the dataset.
(0.04 MB DOC)

Table S5 Distance constraints used for peptide binding prediction and distances for placing the S-PSSMs on the protein surface when scanning for residue binding sites. The table on the left shows the average distance of the CA of each residue from its active center as defined in Table S2 and the table on the right shows the average distance of the CAs of the residues depending on their in-between distance.
(0.05 MB DOC)
