# Supplementary figures and images for: Accurate Prediction of Peptide Binding Sites on Protein Surfaces
Source: PLoS Comput Biol. 2009 Mar 27;5(3):e1000335. doi: 10.1371/journal.pcbi.1000335 (PMC2653190; doi:10.1371/journal.pcbi.1000335)

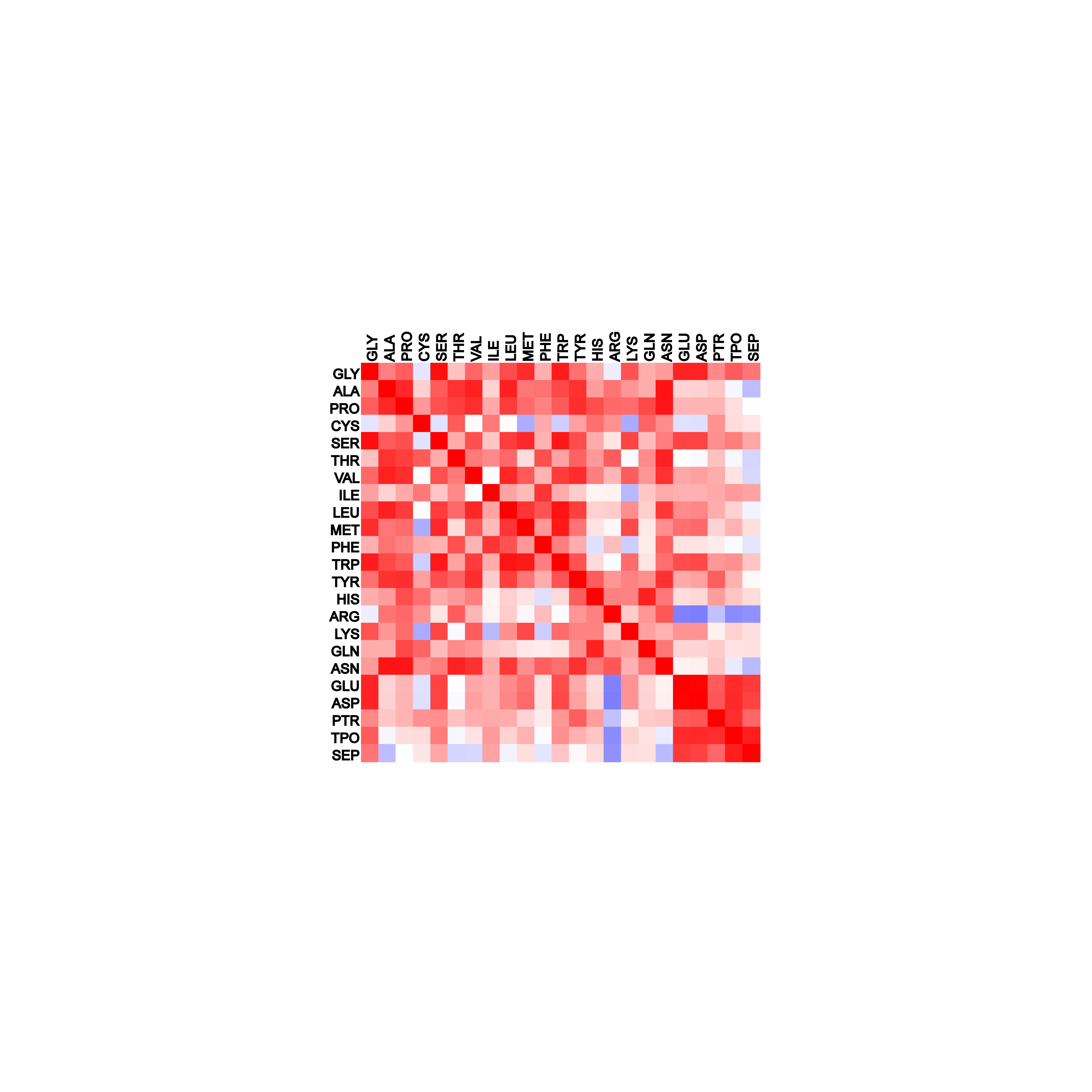

Supplement: Figure S1 — Comparison of S-PSSMs for the 20 standard amino-acid residues and phosphorylated tyrosine (PTR), threonine (TPO), and serine (SEP). Similarities increase from blue to red. (3.25 MB TIF) [file pcbi.1000335.s002.tif]

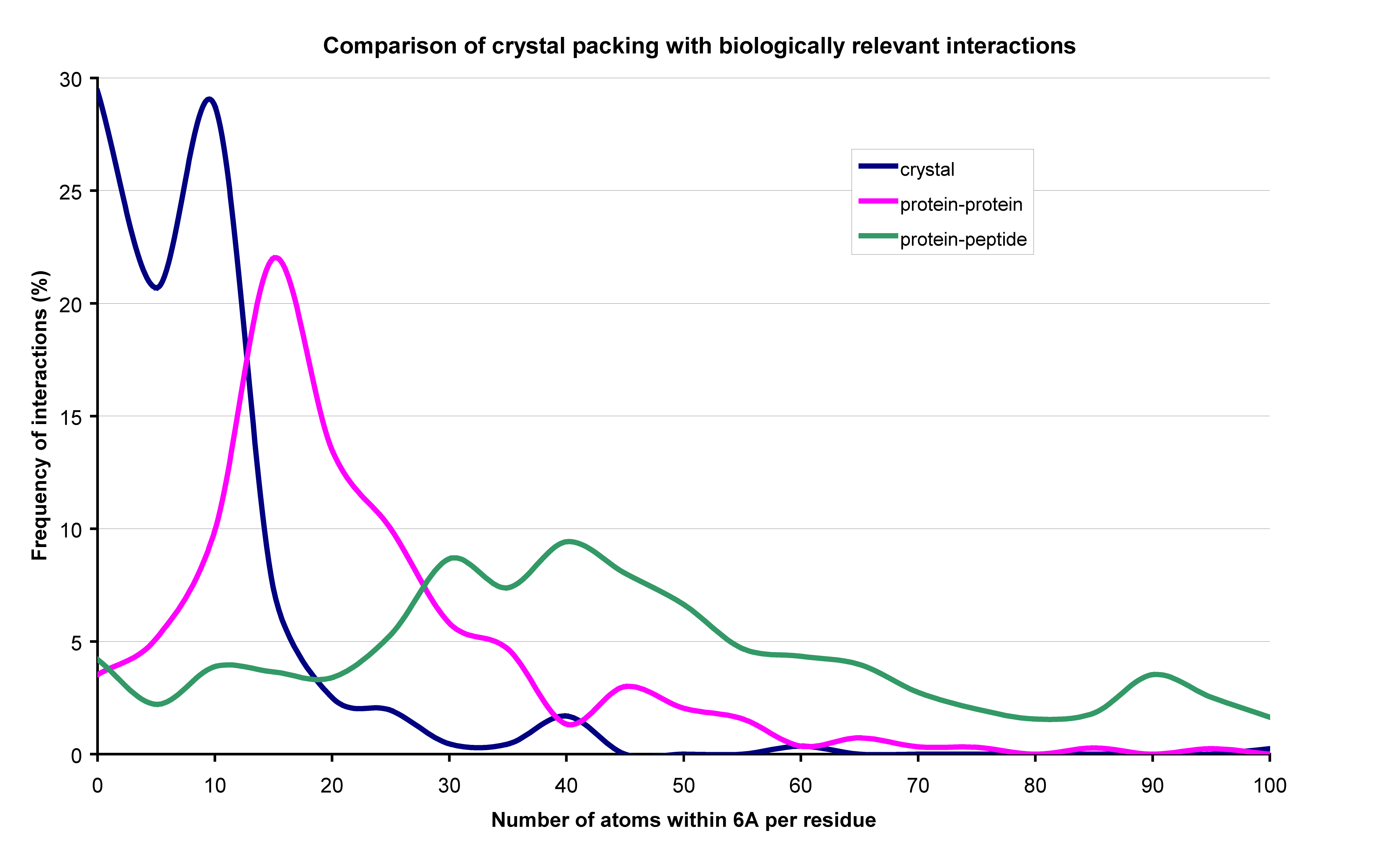

Supplement: Figure S2 — Graph to distinguish protein-peptide interactions from probable crystal packing effects. Distribution of the number of protein atoms within 6 Å of those in the peptide for the three categories: (1) protein-peptide complexes; (2) protein-protein interactions where the interaction consists largely of a peptide stretch from one binding to a globular segment in the other; and (3) probable crystal packing artifacts. (0.75 MB TIF) [file pcbi.1000335.s003.tif]

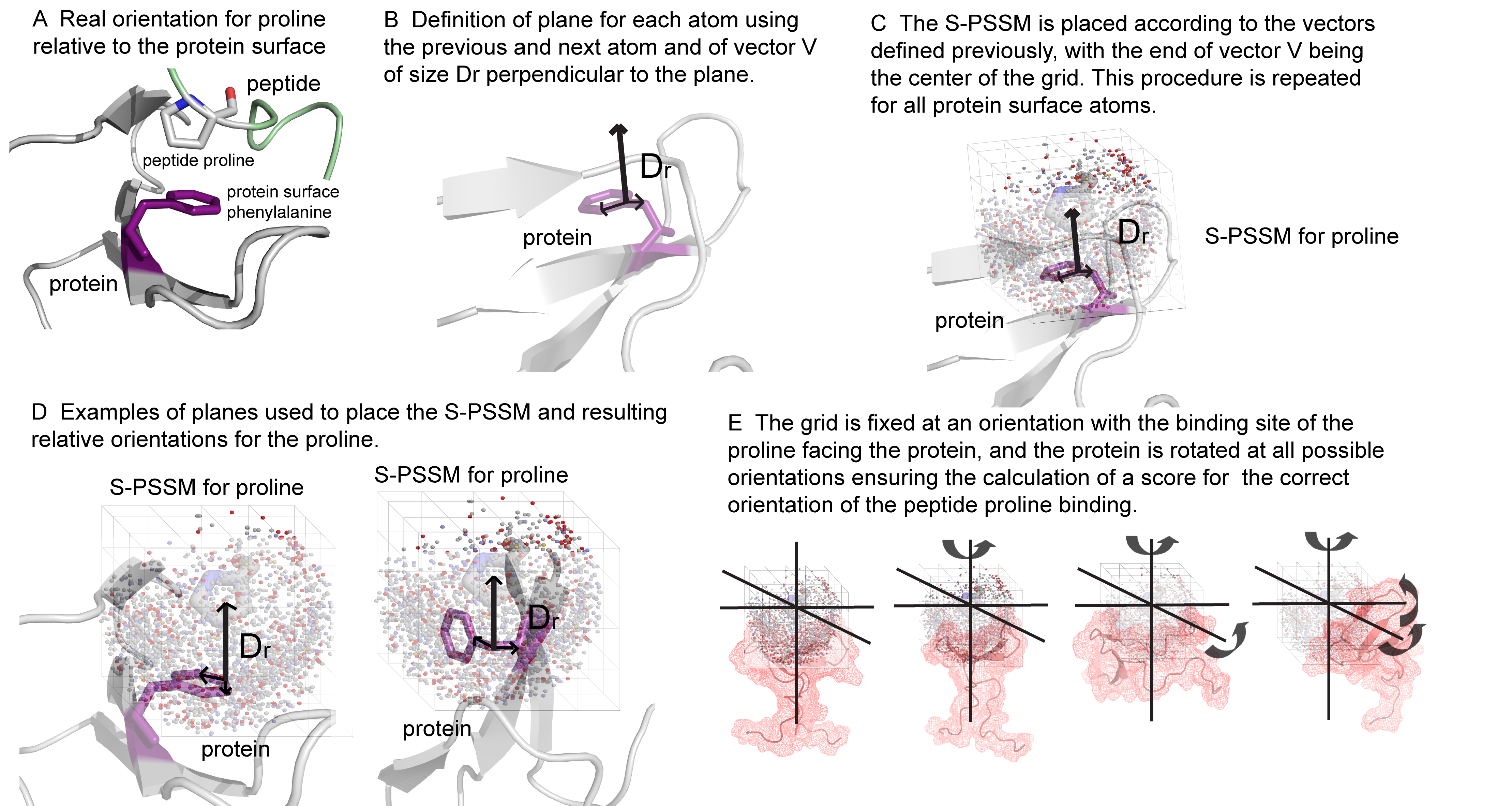

Supplement: Figure S3 — The algorithm for scanning and scoring protein surfaces using the S-PSSMs. (A) Example of real orientation for proline within a peptide relative to a phenylalanine residue on a protein surface. (B) Definition of orientation for the S-PSSM. For each atom of a protein surface a plane is defined using the atoms before and after it in the coordinate file. A vector of distance Dr is then defined to be perpendicular to this plane. (C) The S-PSSM is placed as defined by the previous vectors. (D) Examples of planes resulting to different orientations of the S-PSSM relative to the protein. (E) In practice and in combination with the flexibility provided by the grid cell size (3 Å) repeating this procedure for all protein surface atoms results in an effective rotation of the protein with respect to the S-PSSM in thousands of orientations. (3.77 MB TIF) [file pcbi.1000335.s004.tif]
