# Supplementary material for: Accurate Prediction of Peptide Binding Sites on Protein Surfaces
Source: PLoS Comput Biol. 2009 Mar 27;5(3):e1000335. doi: 10.1371/journal.pcbi.1000335 (PMC2653190; doi:10.1371/journal.pcbi.1000335)
Supplement: Table S1 — Number of complexes per amino acid dataset. (0.03 MB DOC) [file pcbi.1000335.s005.doc]

| **Residue** | **Number of complexes** | **Residue** | **Number of complexes** |
| --- | --- | --- | --- |
| **ARG** | **220** | **ASP** | **221** |
| **GLN** | **160** | **THR** | **205** |
| **PHE** | **216** | **CYS** | **67** |
| **TYR** | **153** | **MET** | **95** |
| **TRP** | **80** | **LEU** | **288** |
| **LYS** | **192** | **ASN** | **135** |
| **GLY** | **208** | **ILE** | **171** |
| **ALA** | **262** | **VAL** | **209** |
| **HIS** | **104** | **TPO** | **13** |
| **SER** | **253** | **SEP** | **17** |
| **PRO** | **246** | **PTR** | **24** |
| **GLU** | **216** |  |  |
