# Supplementary material for: Accurate Prediction of Peptide Binding Sites on Protein Surfaces
Source: PLoS Comput Biol. 2009 Mar 27;5(3):e1000335. doi: 10.1371/journal.pcbi.1000335 (PMC2653190; doi:10.1371/journal.pcbi.1000335)
Supplement: Table S2 — Atoms from the active center of each peptide residue, used for the superimposition of the peptide residue binding sites. TYS was considered equivalent to PTR. Atoms marked with a * represent atoms are not part of the active site but used because the software requires minimum 3 atoms to perform the superimposition (0.04 MB DOC) [file pcbi.1000335.s006.doc]

| **Residue** | **Atoms in active center of residue** | **Residue** | **Atoms in active center of residue** |
| --- | --- | --- | --- |
| **ARG** | **NH2,NH1,NE** | **ASP** | **CG,OD1,OD2** |
| **GLN** | **OE1,NE2,CG*** | **THR** | **CB,OG1,CG2** |
| **PHE** | **CE1,CD1,CG,CD2,CE2,CZ** | **CYS** | **SG, CA*,CB*** |
| **TYR** | **OH,CE1,CZ,CE2,CD2,CG,CD1** | **MET** | **CE,SD,CG** |
| **TRP** | **CG,CD1,CD2,CE2,NE1,CZ2,CZ3,CE3** | **LEU** | **CB,CG,CD1,CD2** |
| **LYS** | **NZ, CD*, CE*** | **ASN** | **CG,ND2,OD1** |
| **GLY** | **CA, N*, C*** | **ILE** | **CD1,CG1,CG2,CB** |
| **ALA** | **CB, C*, CA*** | **VAL** | **CG1,CG2,CB** |
| **HIS** | **CG,ND1,CE1,NE2,CD2** | **TPO** | **P,O1P,O2P,C,O** |
| **SER** | **OG,CA*,CB*** | **SEP** | **P,O1P,O2P,O3P** |
| **PRO** | **CA,CG,CB,CD,N** | **PTR** | **P,O1P,O2P,O3P,CZ,CG,CD2,OH** |
| **GLU** | **CD,OE1,OE2** | **TYS** | **S,O1,O2,O3,CZ,CG,CD2,OH** |
