# Supplementary material for: Accurate Prediction of Peptide Binding Sites on Protein Surfaces
Source: PLoS Comput Biol. 2009 Mar 27;5(3):e1000335. doi: 10.1371/journal.pcbi.1000335 (PMC2653190; doi:10.1371/journal.pcbi.1000335)
Supplement: Table S3 — Atom types and their corresponding atoms in the coordinate files. The different atom types were generated based on their properties. (0.03 MB DOC) [file pcbi.1000335.s007.doc]

| **Atom type** | **Atoms in residues** | **Atom type** | **Atoms in residues** |
| --- | --- | --- | --- |
| **C3**  **(aliphatic carbons; sp3)** | **All CA, All CB, 'CG GLN', 'CG LYS' , 'CG PRO','CG GLU' , 'CG MET' , 'CG LEU' , 'CG1 ILE' ,'CG2 ILE' , 'CG2 THR' , 'CD PRO' ,'CD1 LEU' ,'CD2 LEU' , 'CG1 VAL' ,'CG2 VAL‘, 'CE MET' , 'CD LYS','CE LYS‘, 'CG ARG','CD ARG‘, 'CD1 ILE'** | **OH**  **(hydroxyl group; sp3)** | **'OG SER' , 'OG1 THR' , 'OH TYR'** |
| **C=**  **(carbonyl carbon; sp2)** | **All C, 'CG ASP' ,'CG ASN‘, 'CD GLU' , 'CD GLN'** | **NarH**  **(aromatic nitrogen with a hydrogen; sp2)** | **'NE1 TRP' , 'NE2 HIS'** |
| **O=**  **(carbonyl oxygen; sp2)** | **All O, 'OD1 ASN‘, 'OE1 GLN'** | **NarH+**  **(aromatic nitrogen with a hydrogen and a postive charge; sp2)** | **'ND1 HIS'** |
| **N2H**  **(nitrogen of amides; sp2; also sp2 neutral nitrogen of side chains)** | **'N ARG' , 'N GLN' ,'N PHE' ,'N TYR' ,'N TRP' ,'N LYS' ,'N GLY' ,'N ALA' ,'N HIS' ,'N SER' ,'N PRO' ,'N GLU' ,'N ASP' ,'N THR' ,'N CYS' ,'N MET' ,'N LEU' ,'N ASN' ,'N ILE' ,'N VAL' , 'NH1 ARG','NH2 ARG‘,'ND2 ASN' , 'NE2 GLN'** | **Set**  **(sulphur in thioethers; sp3)** | **'SD MET'** |
| **Car**  **(aromatic carbon; sp2; general)** | **'CG PHE' ,'CG TYR' ,'CG TRP' ,'CG HIS‘, 'CD1 TRP' ,'CD2 TRP' , 'CE2 TRP' ,'CE3 TRP‘ ,'CZ2 TRP' ,'CZ3 TRP' ,'CH2 TRP' , 'CD1 TYR' ,'CD2 TYR' ,'CE1 TYR' ,'CE2 TYR' ,'CZ TYR‘, 'CD2 HIS' , 'CD1 PHE' ,'CD2 PHE' ,'CE1 PHE' ,'CE2 PHE' ,'CZ PHE' , 'CE1 HIS'** | **C+**  **(carbon of carbocations; sp2)** | **'CZ ARG'** |
| **O2-**  **(negatively charged oxygens (-1/2) in carboxylates; sp2)** | **All OXT, 'OD1 ASP' ,'OD2 ASP‘, 'OE2 GLU' ,'OE1 GLU'** | **N3H+**  **(sp3 nitrogen with a hydrogen and a positive charge)** | **'NZ LYS'** |
| **SH**  **(sulphur in thiols; sp3)** | **'SG CYS'** | **N2H+**  **(sp2 nitrogen with a hydrogen and a positive charge)** | **'NE ARG'** |
