# Supplementary material for: Accurate Prediction of Peptide Binding Sites on Protein Surfaces
Source: PLoS Comput Biol. 2009 Mar 27;5(3):e1000335. doi: 10.1371/journal.pcbi.1000335 (PMC2653190; doi:10.1371/journal.pcbi.1000335)
Supplement: Table S4 — Range of scores per residue hot-spots. The table shows minimum value, average value, standard deviation, and maximum value observed for the dataset. (0.04 MB DOC) [file pcbi.1000335.s008.doc]

| **Residue** | **Min/avg/stdev/max** | **Residue** | **Min/avg/stdev/max** |
| --- | --- | --- | --- |
| **ARG** | **1 / 8.9438 / 3.251 / 28** | **ASP** | **1 / 8.4893 / 3.354 /32** |
| **GLN** | **5 / 14.213 / 4.52 / 39** | **THR** | **1 / 9.5875 / 3.699 / 28** |
| **PHE** | **1 / 9.6031 / 3.3 / 28** | **CYS** | **-1 / 19.731 / 6.765 /50** |
| **TYR** | **3 / 9.3899 / 3.145 / 25** | **MET** | **-2 / 11.920 / 3.301 /35** |
| **TRP** | **5 / 12.037 / 3.415 / 27** | **LEU** | **-2 / 8.8788 / 2.385 /27** |
| **LYS** | **-2 / 8.8221 / 3.464 / 28** | **ASN** | **1 / 11.673 / 3.613 / 34** |
| **GLY** | **1 / 7.9240 / 3.489 / 25** | **ILE** | **2.5 / 11.529 / 3.361 / 28** |
| **ALA** | **0 / 9.8132 / 4.229 / 32** | **VAL** | **1/ 9.7950 / 3.122 / 26** |
| **HIS** | **5 / 15.710 / 5.203 / 46** | **TPO** | **25 / 38.454 / 8.799 / 83** |
| **SER** | **-2 / 6.9320 / 3.415 / 28** | **SEP** | **18 / 30.274 / 4.803 / 57** |
| **PRO** | **-1 / 17.181 / 7.278 /50** | **PTR** | **-2 / 25.252 / 5.554 / 53** |
| **GLU** | **2 / 8.2041 / 2.533 /25** |  |  |
