# Supplementary material for: Accurate Prediction of Peptide Binding Sites on Protein Surfaces
Source: PLoS Comput Biol. 2009 Mar 27;5(3):e1000335. doi: 10.1371/journal.pcbi.1000335 (PMC2653190; doi:10.1371/journal.pcbi.1000335)
Supplement: Table S5 — Distance constraints used for peptide binding prediction and distances for placing the S-PSSMs on the protein surface when scanning for residue binding sites. The table on the left shows the average distance of the CA of each residue from its active center as defined in Table S2 and the table on the right shows the average distance of the CAs of the residues depending on their in-between distance. (0.05 MB DOC) [file pcbi.1000335.s009.doc]

| **Residue** | **Distance R-CA D_r_ (Å)** |  | **Residue** | **Distance**  **D_Cal_ (Å)** |
| --- | --- | --- | --- | --- |
| **ARG** | **3.051** |  | **0** | **0** |
| **GLN** | **4.753** |  | **1** | **4.501** |
| **PHE** | **4.162** |  | **2** | **6.697** |
| **TYR** | **4.261** |  | **3** | **8.442** |
| **TRP** | **4.115** |  | **4** | **10.148** |
| **LYS** | **6.104** |  | **5** | **12.081** |
| **GLY** | **1.234** |  | **6** | **13.532** |
| **ALA** | **2.010** |  | **7** | **14.693** |
| **HIS** | **4.628** |  | **8** | **16.116** |
| **SER** | **2.734** |  | **9** | **17.267** |
| **PRO** | **1.733** |  | **10** | **18.157** |
| **GLU** | **4.248** |  | **11** | **19.057** |
| **ASP** | **3.051** |  | **12** | **19.942** |
| **THR** | **2.479** |  | **13** | **24.095** |
| **CYS** | **3.064** |  | **14** | **21.543** |
| **MET** | **3.632** |  | **15** | **21.500** |
| **LEU** | **3.311** |  | **16** | **21.772** |
| **ASN** | **2.973** |  | **17** | **21.974** |
| **ILE** | **2.822** |  | **18** | **21.554** |
| **VAL** | **2.506** |  | **19** | **20.263** |
| **TPO** | **3.890** |  | **20** | **18.893** |
| **SEP** | **4.590** |  | **21** | **18.594** |
| **PTR** | **6.600** |  | **22** | **18.376** |
